# Association of CYP3A4-392A/G, CYP3A5-6986A/G, and ABCB1-3435C/T Polymorphisms with Tacrolimus Dose, Serum Concentration, and Biochemical Parameters in Mexican Patients with Kidney Transplant

**DOI:** 10.3390/genes15040497

**Published:** 2024-04-16

**Authors:** Edith Viridiana Alatorre-Moreno, Ana Miriam Saldaña-Cruz, Edsaúl Emilio Pérez-Guerrero, María Cristina Morán-Moguel, Betsabé Contreras-Haro, David Alejandro López-de La Mora, Ingrid Patricia Dávalos-Rodríguez, Alejandro Marín-Medina, Alicia Rivera-Cameras, Luz-Ma Adriana Balderas-Peña, José Juan Gómez-Ramos, Laura Cortés-Sanabria, Mario Salazar-Páramo

**Affiliations:** 1 Centro Universitario de Ciencias de la Salud, Departamento de Nefrología, Hospital de Especialidades, Centro Médico Nacional de Occidente, Instituto Mexicano del Seguro Social, Universidad de Guadalajara, Guadalajara 44340, Mexico; ediviridiana@hotmail.com; 2 Centro Universitario de Ciencias de la Salud, Departamento de Fisiología, Instituto de Terapéutica Experimental y Clínica, Universidad de Guadalajara, Guadalajara 44340, Mexico; ana.saldanac@academicos.udg.mx; 3 Centro Universitario de Ciencias de la Salud, Instituto de Investigación en Ciencias Biomédicas, Universidad de Guadalajara, Guadalajara 44340, Mexico; edsaul.perezg@academicos.udg.mx; 4 Departamento de Biología Molecular y Genómica, Centro Universitario de Ciencias de la Salud, Universidad de Guadalajara, Guadalajara 44340, Mexico; cristina.moran@academicos.udg.mx (M.C.M.-M.); alejandro.marin@academicos.udg.mx (A.M.-M.); 5 Departamento de Ciencias Biomédicas, Centro Universitario de Tonalá, UIB02, Hospital de Especialidades, Centro Médico Nacional de Occidente, Instituto Mexicano del Seguro Social, Universidad de Guadalajara, Guadalajara 44340, Mexico; betsabecoha@gmail.com; 6 Departamento de Ciencias Biomédicas, Centro Universitario de Tonalá, Universidad de Guadalajara, Guadalajara 44340, Mexico; phd.delamora@gmail.com; 7 Departamento de Biología Molecular y Genómica, División de Genética, Centro de Investigación Biomédica de Occidente, Centro Universitario de Ciencias de la Salud, Instituto Mexicano del Seguro Social, Universidad de Guadalajara; Guadalajara 44340, Mexico; ingrid.davalos@academicos.udg.mx (I.P.D.-R.); alicia.rivera3492@alumnos.udg.mx (A.R.-C.); 8 Departamento de Morfología, Centro Universitario de Ciencias de la Salud, UIB02, Hospital de Especialidades Centro Médico Nacional de Occidente, Instituto Mexicano del Seguro Social, Universidad de Guadalajara, Guadalajara 44340, Mexico; luz.balderas@academicos.udg.mx; 9 Departamento de Urgencias, Hospital General de Zona 89, Instituto Mexicano del Seguro Social, Guadalajara 44340, Mexico; josejuan79@yahoo.com; 10 Centro Médico Nacional de Occidente, Hospital de Especialidades, Instituto Mexicano del Seguro Social, Guadalajara 44340, Mexico; cortes_sanabria@yahoo.com.mx; 11 Departamento de Fisiología, Centro Universitario de Ciencias de la Salud, Academia de Inmunología, Universidad de Guadalajara, Guadalajara 44340, Mexico

**Keywords:** tacrolimus, SNPs, pharmacokinetics, kidney transplant recipients

## Abstract

Tacrolimus (TAC) is an immunosuppressant drug that prevents organ rejection after transplantation. This drug is transported from cells via P-glycoprotein (ABCB1) and is a metabolic substrate for cytochrome P450 (CYP) 3A enzymes, particularly CYP3A4 and CYP3A5. Several single-nucleotide polymorphisms (SNPs) have been identified in the genes encoding *CYP3A4*, *CYP3A5*, and *ABCB1*, including CYP3A4-392A/G (rs2740574), CYP3A5 6986A/G (rs776746), and ABCB1 3435C/T (rs1045642). This study aims to evaluate the association among CYP3A4-392A/G, CYP3A5-6986A/G, and ABCB1-3435C/T polymorphisms and TAC, serum concentration, and biochemical parameters that may affect TAC pharmacokinetics in Mexican kidney transplant (KT) patients. Methods: Forty-six kidney transplant recipients (KTR) receiving immunosuppressive treatment with TAC in different combinations were included. CYP3A4, CYP3A5, and *ABCB1* gene polymorphisms were genotyped using qPCR TaqMan. Serum TAC concentration (as measured) and intervening variables were assessed. Logistic regression analyses were performed at baseline and after one month to assess the extent of the association between the polymorphisms, intervening variables, and TAC concentration. Results: The GG genotype of CYP3A5-6986 A/G polymorphism is associated with TAC pharmacokinetic variability OR 4.35 (95%CI: 1.13–21.9; *p* = 0.0458) at one month of evolution; in multivariate logistic regression, CYP3A5-6986GG genotype OR 9.32 (95%CI: 1.54–93.08; *p* = 0.028) and the use of medications or drugs that increase serum TAC concentration OR 9.52 (95%CI: 1.79–88.23; *p* = 0.018) were strongly associated with TAC pharmacokinetic variability. Conclusion: The findings of this study of the Mexican population showed that CYP3A5-6986 A/G GG genotype is associated with a four-fold increase in the likelihood of encountering a TAC concentration of more than 15 ng/dL. The co-occurrence of the CYP3A5-6986GG genotype and the use of drugs that increase TAC concentration correlates with a nine-fold increased risk of experiencing a TAC at a level above 15 ng/mL. Therefore, these patients have an increased susceptibility to TAC-associated toxicity.

## 1. Introduction

Chronic kidney disease (CKD) is a significant public health problem worldwide, with increasing incidence [1,2] In Mexico, according to the Renal Data System of the United States 2022 report, the incidence rate is 274 patients per million inhabitants, which is the eighth highest in the world [3].

The most serious manifestation of CKD is end-stage renal disease (ESRD), which is characterized by the presence of severe and irreversible kidney damage that results in a glomerular filtration rate of less than 15 mL/min, leading to a clinical condition known as uremia [4]. The primary treatment for ESRD is renal replacement therapy (RRT), which includes peritoneal dialysis (PD), hemodialysis (HD), or kidney transplantation (KT) [5]. One of the mainstays of treatment after KT is immunosuppression, which must be individualized and tailored to each patient. Currently, the most widely used regimen consists of tacrolimus (TAC), mycophenolic acid (MPA), and prednisone (PDN), immunosuppressive treatment for KT involves thymoglobulin or basiliximab for initial induction. For maintenance, anti-rejection agents include prednisone, mycophenolate, TAC, or cyclosporine [6,7].

TAC, also known as FK-506, is a macrolide that binds to an immunophilin known as tacrolimus binding protein 12 (FKBP12). This protein, in turn, binds to calcineurin and inhibits the phosphatase domain that facilitates the migration of NF-AT (nuclear factor of activated T cells) from the cytoplasm to the nucleus. This process promotes the transcription of the interleukin-2 gene and other cytokines, thereby blocking the proliferation and function of T lymphocytes [8,9]. At the intestinal concentration, TAC acts as a substrate for P-glycoprotein (PgP), which the *ABCBI* gene encodes. TAC is primarily metabolized in the liver by the cytochrome P450 enzyme system, specifically by reductases of the CYP3A5 (60%) and CYP3A4 (40%) families, which are also present in the intestine but to a lesser extent. Demethylation and hydroxylation are the main metabolic pathways. For renal replacement, TAC ranges may vary according to KT time: ≤30 days, 10–15 ng/mL; 1 to 3 months, 10–12 ng/mL; 4 to 6 months, 8–10 ng/mL; 6 to 12 months, 6–10 ng/mL; less than 12 months, 6–8 ng/mL [10].

More than 15 metabolites have been isolated from plasma, urine, and bile [11,12]. TAC exhibits significant inter- and intra-individual pharmacokinetic variability, requiring close monitoring of its serum concentration to avoid rejection and toxicity. Genetic factors may account for 20 to 95% of the pharmacokinetic variability of TAC. Demographic factors and drug–drug interactions also contribute to patient variability in TAC pharmacokinetics [13]. 

It has been reported that genetic polymorphisms, clinical factors, and medication accounted for TAC concentration variability in patients with KT [14].

Genetic factors (SNPs) in the *CYP3A4*, *CYP3A5*, and *ABCB1* genes have been observed to influence TAC pharmacokinetics [15,16]. The CYP3A4-392A/G (rs2740574), CYP3A5-6986A/G (rs776746), and ABCB1-3435C/T (rs1045642) polymorphisms are the genetic factors most associated with TAC pharmacokinetic variability (over- or under-exposure). Genotype frequencies have an important role in the clinical validity of genomic profiling and the number of individuals who are at increased risk. Genotype frequencies should therefore be given greater attention when reporting the results of association studies [17].

Genetic variability in the Mexican population has been reported. Polymorphisms studies in particular populations are relevant, which could imply a diverse drug response, and there is limited information regarding these polymorphisms and TAC in Mexican KT patients [18,19]. Although the results vary depending on the population studied, genotyping could be useful for the dosing approach and therapeutic drug monitoring in the post-transplant phase [19,20,21,22,23,24,25]. The allele frequencies (AF) in Mexican population have been reported for CYP3A4-392A/G, A 0.96, G 0.04, for CYP3A5-6986A/G, A 0.18, G 0.82 and ABCB1-3435C/T, C 0.52, T 0.48 [24].

This study aimed to evaluate the association among CYP3A4-392 A/G (rs2740574), CYP3A5-6986 A/G (rs776746), and ABCB1-3435 C/T (rs1045642) polymorphisms with TAC concentration, TAC doses and biochemical parameters that may impact the TAC pharmacokinetics in Mexican patients with KTR.

## 2. Materials and Methods

### 2.1. Study Population

Forty-six Mexican patients with KT were selected from the Departamento de Nefrología, Centro Médico Nacional de Occidente (CMNO) IMSS, Guadalajara, Mexico.

This study included a prospective cohort of KT Mexican patients (self-reported of at least three generations of Mexican ancestry), age >18 years, any gender, incident KT and receiving TAC immunosuppressive treatment in any combination and with any donor type. Patients diagnosed with conditions affecting gastrointestinal absorption or motility (such as intestinal obstruction and diabetic gastroparesis), those with liver disease, and recipients of combined transplants (liver and kidney) were excluded.

On day two after transplantation, TAC was administered orally under fast, starting with a dosage between 3 and 5 mg twice daily. The basal TAC concentration (measured in whole blood) and TAC dose were evaluated on the fifth day after transplantation and twice weekly afterward for suspicion of graft rejection or adverse events. TAC troughs were measured 12 h after the last dose was administered at a steady-state concentration of the last administration, and the dose was adjusted to achieve the therapeutic window target concentrations after a month of follow-up. Other biochemical parameters were measured at basal and after one-month follow-up.

### 2.2. Polymorphisms Genotyping

Genomic DNA was extracted from the blood samples using the modified Miller technique [26]. Genotyping of CYP3A4-392A/G (rs2740574), CYP3A5-6986A/G (rs776746), and ABCB1-3435C/T (rs1045642) polymorphisms were performed via real-time quantitative polymerase chain reaction (qPCR) using TaqMan predesigned probes. TaqMan assays with IDs C_1837671_50, C_26201809_30, and C_7586657_20 was used. The qPCR assay was performed using StepOneTM system (Applied Biosystems, Thermo Fisher Sci, Waltham, MA, USA) according to the manufacturer’s recommendations.

### 2.3. TAC Quantification and Biochemical Parameter Determination

Venous blood samples were obtained to quantify TAC levels and other biochemical parameters. TAC concentration was measured by the microparticle chemiluminescent immunoassay analytical method and equipment Architect^®^ (Abbott, Chicago, IL, USA). The therapeutic window was established between 5.0 and 15.0 ng/mL; levels higher than 15 ng/mL were considered toxic [10,27,28]. TAC concentration was established, representing the threshold indicating the most significant risk of initial-month toxicity due to TAC belongs methods. Other chemical parameters—hematocrit, albumin, and creatinine—were measured via an enzymatic colorimetric method in a VITROS 4600^®^ (Ortho Clinical Diagnostics, Raritan, NJ, USA). We report a month follow up one-time TAC measurement because that is enough time to evaluate significant modifications that could reflect changes in TAC pharmacokinetics like renal function, albumin concentration, dose-reduction of steroids, antihypertensive drugs, and inhibitor pump inhibitors use [29]. 

### 2.4. Pharmacogenetic Analysis

The genetic profile was constructed from the allelic frequency and analyzed for their association with response to treatment using an SNP analyzer.

### 2.5. Statistical Analysis

Data analysis was performed using the R programming language for statistical analysis, version 4.2. Quantitative variables with parametric distribution were presented as means ± standard deviation, and those with non-parametric distribution as medians and ranges (min–max). Dependent samples T-test (normal distribution) and the Wilcoxon test (non-parametric distribution) were performed to compare baseline and one-month evolution for serum TAC concentration, TAC doses, and intervening variables The U-Mann–Whitney U test, Kruskal–Wallis test, Student’s *t*-test, or the one-way ANOVA test was employed to compare serum TAC concentration across genotypes for each polymorphism. A logistic regression model was performed in which the independent variable was low TAC levels (<15 ng/dL). The model was adjusted by variables that in the bivarized bivariate analysis (<15 ng/dL vs. >15 ng/dL) had a *p*-value lower than 0.20 or by those variables with biological plausibility. In the final model, three genetic variants were studied (CYP3A4-392A/G, CYP3A5-6986A/G, ABCB1-*3435C*/T), as well as serum creatinine levels, hematocrit after 1 month, and the use of drugs that raise TAC concentrations. The stepwise method was utilized to select the final model. We computed the odds ratio (OR) and their 95% confidence intervals (95%CI). A *p*-value of <0.05 was considered as indicative of significance. 

## 3. Results

### 3.1. Demographic and Clinical Features of the Study Population

Forty-six incidental KT patients undergoing TAC under immunosuppressive treatment were enrolled. The mean age of the patients was 29.8 years (29.8 ± 5.3); 80% were male. The median duration of CKD diagnosis was four years, and the etiology of CKD was unknown in more than 90% of the patients. High blood pressure was the most common comorbidity, affecting 83% of patients.

The demographic and clinical features of the study population are shown in Table 1.

### 3.2. Analysis of the SNPs: CYP3A4-392A/G, CYP3A5-6986A/G, and ABCB1-3435C/T Polymorphisms

The CYP3A4-392A/G polymorphism was identified in 45 patients. The most prevalent genotype was AA, observed in 82% of the patients. None of the patients exhibited the GG genotype. The most common allele was the wild-type allele (A), present in 91% of the patients; *CYP3A5-6986A/G* polymorphism was identified in 46 patients. The GG genotype was observed in 63% of the patients. The predominant allele was the variant allele (G), present in 82% of the patients. None of the studied patients exhibited the AA genotype. The prevalent allele was the variant allele (G) in 82% of the patients. The *ABCB1-3435C/T* polymorphism was identified in 44 patients. The most prevalent genotype was CT, observed in 66% of the patients. The prevalent allele was the variant allele (T), present in 60% of the patients.

As to the genetic profile of CYP3A4-392A/G, CYP3A5-6986A/G, and ABCB1-3435C/T polymorphisms, nine of the genetic profiles from three SNPs generated from the combinations from the genotyping were analyzed. Only four were identified with a frequency higher than 5% from these combinations. These four combined genotypes were present in 90% of all patients. The genetic profile more frequently found was 1 (GAT) at 50% following 2 (GAC) at 26%. The genotype and allele frequencies and genetic profile of the study population for the *CYP3A4*-392A/G, *CYP3A5*-6986A/G, and *ABCB1*-3435C/T polymorphisms are shown in Table 2.

### 3.3. CYP3A4-392A/G, CYP3A5-6986A/G, and ABCB1-3435C/T Genotypes, TAC Concentration, and Dose

The genotypes were compared with the levels and doses of basal TAC at the baseline and one month after transplant. At the baseline concentration, no statistically significant difference was identified between the genotypes for either of the two variables However, after one-month follow-up, a significant difference was shown between the genotypes for the CYP3A5-6986A/G polymorphism and the TAC levels (*p =* 0.006). These findings are shown in Table 3.

### 3.4. TAC Concentration and Genetic Profile

**Figure 1:**
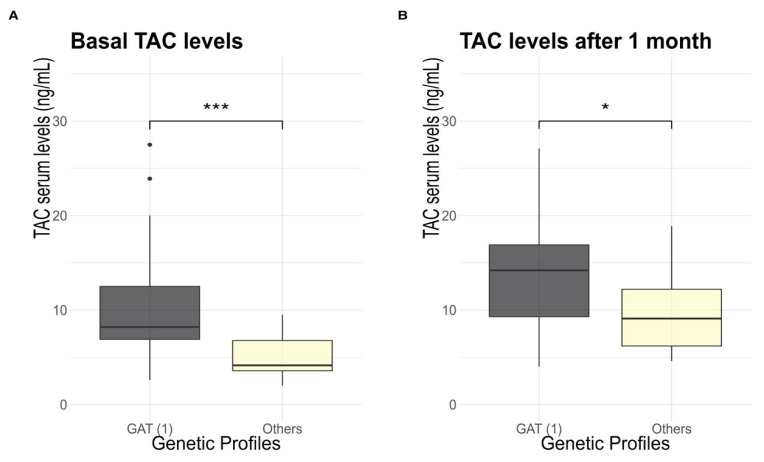
(**A**) Comparison between TAC levels and genetic profile in baseline; *p* = 0.009. (**B**) Comparison between TAC levels and genetic profile after one month of kidney transplantation measurements; *p* = 0.04. * means *p* < 0.05; *** means *p* < 0.001.

TAC concentration was also assessed based on the different genetic profiles (Figure 1). For the CYP3A5-6986-A/G variant, a remarkable distinction in TAC concentration was identified when comparing different genetic profiles. 

### 3.5. Comparison of TAC Concentration TAC Dose, Biochemical Parameters, and Concurrent Drugs at Baseline and One Month after Evolution

In comparison to their baseline measurements in KTR patients, it was observed that after one month of treatment, the serum TAC median concentration increased from 8.1 (range: 5.0–11.8) to 13.5 ng/mL (range: 9.0–16.18), hematocrit concentration from 32.9 (±6.7) to 39.5% (±4.4). The median serum creatinine concentration decreased from 1.3 (range: 0.47–1.97) to 1.01 (range: 0.11–013) mg/dL.

The percentage of concurrent drugs that impact TAC’s pharmacokinetics decreased from 65% to 39% (these drugs were omeprazole, amlodipine, or the omeprazole-nifedipine combination). Conversely, serum albumin and TAC dosage remained unchanged. These outcomes are detailed in Table 4.

### 3.6. Assessment of the Association of Genetic and Biochemical Parameters with TAC Concentration

Consequently, patients were categorized into two groups: those with TAC ≤15 ng/mL (group 1) and those with TAC >15 ng/mL (group 2). A comparison of the main characteristics between both groups is presented in Table 5. At baseline determination, TAC in group 1 was 7.35 ng/mL and 21.95 ng/mL in group 2, with no significant distinction observed between genetic and biochemical parameters (creatinine serum, hematocrit, serum albumin, hypoalbuminemia).

One month after progression, the TAC median concentration level in group one was 10.02 ng/mL, while group two exhibited 18.18 ng/mL as median. The frequency of CYP3A5-6986A/G genotypes in both groups displayed statistical significance, with *p* < 0.05. The CYP3A5-6986 GG genotype frequency was similar in both groups, whereas the AG genotype was more prevalent in group one. Furthermore, serum creatinine demonstrated a significant difference between the two groups, being higher in group one. Serum albumin also exhibited a significant difference between the groups; however, it is essential to highlight that none of the patients presented hypoalbuminemia (serum albumin <3.5 g/L) after a month of progression (Table 5). 

To assess the magnitude of the association between the polymorphisms and intervening variables, a logistic regression analysis was conducted for both baseline and one-month-later variables. In the baseline logistic regression model, none of the variables demonstrated individual or collective significance. For the analysis one month later, the variables showing a notable difference compared to their baseline measurements were integrated. The presence of the GG genotype of the CYP3A5-6986A/G polymorphism showed an OR of 4.35 (95%CI: 1.13–21.90; *p* = 0.0458) for serum TAC concentration > 15 ng/dL at one month of evolution. In a combined analysis with the remaining included variables, the GG genotype of the CYP3A5-6986-A/G polymorphism demonstrated an OR of 9.32 (95%CI: 1.54–93.08; *p* = 0.028), while the drug increasing serum TAC level presented an OR of 9.53 (95%CI: 1.79–88.23; *p* = 0.018). The attributes of logistic regression models are outlined in Table 6.

## 4. Discussion

The influence of genetic factors on the pharmacokinetic variability of TAC appears to be significant. In this study, we evaluated the relationship between CYP3A4-392A/G, CYP3A5-6986A/G, and ABCB1-3435C/T polymorphisms with TAC concentration in KTR. We identified an association between the CYP3A5-6986A/G polymorphism and TAC pharmacokinetic variability. Using an adjusted logistic regression analysis, we determined that the presence of CYP3A5-6986A/G increases the risk of TAC concentration. This increased risk of TAC levels (>15 ng/mL) was independent from other factors, including biochemical parameters like hematocrit, serum creatinine, and some known drugs that increase TAC concentration. Although this association has been reported in other populations [16,30,31,32], the genetic variability among different racial groups, and polymorphisms studies in particular populations, are relevant, with implications for drug response. To our knowledge, there is limited information regarding these polymorphisms and TAC concentration in Mexican KT patients [18,19].

We observed that patients with the CYP3A5-6986GG genotype had elevated serum TAC concentration compared to heterozygotes (AG). This difference is because patients with the GG genotype have a slower metabolism than heterozygotes, which serve as intermediate metabolizers. This phenomenon is related to the biological effect of the A-to-G substitution, which leads to a splicing defect in the mRNA. This modification generates an unstable and non-functional protein, which ultimately affects the TAC concentration in these patients [32,33,34]. The genetic profile encompassing the GG genotype of CYP3A5-6986A/G showed a significantly elevated of concentration TAC when contrasted with those with the AG genotype.

Regarding the other polymorphisms analyzed, CYP3A4-392A/G, CYP3A5-6986A/G, and ABCB1-3435C/T, our study did not show a significant difference between genotypes and serum TAC concentration. The associations between these polymorphisms and serum TAC are concentration inconsistent, probably due to the specific population studied [35,36,37].

In terms of overall population characteristics, most patients were men, and the underlying etiology of CKD remained undetermined in a considerable proportion of cases; this has been reported in other studies in the Mexican population [38,39].

Other factors influence TAC pharmacokinetic variability, particularly liver dysfunction, gastrointestinal motility disorders, food and/or drug interactions, hematocrit, albumin concentration, and renal function [40].

In this study, the biochemical parameters evaluated were hematocrit, serum albumin, serum creatinine, and drug interactions. Hematocrit and serum creatinine concentration showed significant changes after one month of evolution, in contrast to baseline measurements (as shown in Table 5). These changes are attributed to the physiological shifts associated with satisfactory renal graft function observed in most subjects. Therefore, while hematocrit may predict TAC concentration variability within whole blood, this may not apply to therapeutically active concentrations [39]. Other studies have documented a substantial correlation between hematocrit and creatinine with concentration *log tacrolimus* [41,42], suggesting the potential impact of these factors on TAC pharmacokinetics. However, in our study, we included genetic and biochemical parameters. We did not observe any divergence in hematocrit and serum creatinine concentration.

In terms of drug interactions, the most commonly co-administered drugs were omeprazole, nifedipine, amlodipine, or the omeprazole-nifedipine combination. After one month of observation, a notable reduction in their use was observed, from 65% to 39%. However, there was a significant increase in TAC concentration after one month, from 8.1 to 13.5 ng/mL. The interaction between TAC and omeprazole which are metabolized in the liver by the CYP3A4 enzyme could be explained by the fact that omeprazole is a CYP2C19 and CYP3A enzyme inhibitor that could modify TAC pharmacokinetics: CYP2C19 inhibition may suppress omeprazole metabolism, leading to metabolic pathway alterations by the CYP3A enzyme to maintain an adequate biotransformation. Subsequently, CYP3A4 competition increases TAC serum concentration [24]. Concomitant administration of these medications may affect TAC pharmacokinetics, particularly in patients with genetic SNPs in CYP3A5 [43,44,45,46]. 

Amlodipine is metabolized in the liver by the CYP3A4 and CYP3A5 enzymes and by PgP. In individuals with SNPs that render the CYP3A5 protein nonfunctional, the CYP3A4 pathway assumes a major role in metabolism. This phenomenon appears to increase TAC concentration in patients taking amlodipine due to potential interactions [47]. Therefore, despite the reduction in omeprazole and amlodipine use during the first month, the TAC concentration increased. Multiple interactions among various drugs in CYP450 are well known, including inductions and inhibitions. However, the chemical structure of CYP450 has a wide site of union to the substrate, suggesting that this enzyme can bind simultaneously to diverse ligands during its biological functions [46].

Consequently, patients with CYP3A5-6986A<G genotype may require close therapeutic monitoring when subjected to combined TAC/omeprazole/amlodipine therapies. 

Therefore, a comprehensive analysis including genetic factors such as the identification of CYP3A5-6986A<G polymorphism and drug interactions in Mexican KT patients may provide a more robust assessment of TAC pharmaceutical variability. This, in turn, may allow for more precise dose adjustments, thereby contributing to preventing toxicity events in this population.

### Study Strengths and Limitations

In the best of our knowledge, this study is the first to assess the relation between CYP3A4-392A/G, CYP3A5-6986A/G, and ABCB1-3435C/T with TAC concentration in KT Mexican patients. Additionally, in our research, using an adjusted logistic regression analysis, we can conclude that the presence of CYP3A5-6986A/G increase the risk of TAC concentration. This increased risk of TAC concentration >15 ng/mL was independent from other factors, including serum creatinine, hematocrit, and drugs that increase tacrolimus concentration. This study in Mexican KT patients opens the possibility of reconsidering the usefulness of establishing an initial TAC dose based on genotype, which could offer benefits not only in reducing the number of dose modifications but also in reducing the rates of nephrotoxicity and KT rejection. 

There are some limitations in our research in relation to the findings of the main effects of individual SNPs and the increase in TAC concentration. First, we cannot exclude the possibility that other gene regions may be important. Second, TAC concentration could be increased by multifactorial causes, and this finding supports the notion that risk factors may be relevant only in a proportion of the population with underlying genetic susceptibility. Future investigations that could replicate the findings in this research are necessary to verify the biological precept of the plausibility of gene–environment interactions as they relate to the genotypes and TAC concentration. Third, a limitation that must be considered in this study is the low statistical power. Therefore, new studies are required in which the sample size could be increased to validate the results shown in this study. Studies in larger and different populations with a longer follow-up focusing on comparing the standard dose of TAC with a genotype-adapted dose are required to evaluate whether genotype determination in KT confers a benefit in terms of outcomes, such as TAC toxicity, rejection, hospitalization time, costs, etc.

## 5. Conclusions

The findings of this study suggest that the CYP3A5-6986A/G GG genotype is associated with a four-fold-increased likelihood of experiencing serum TAC concentration greater than 15 ng/mL after one month of KT. Co-occurrence of the CYP3A5-6986A/G GG genotype and use of TAC-increasing drugs correlates with a nine-fold-increased susceptibility to increased TAC concentration exceeding 15ng/mL one month after KT. Therefore, close monitoring of these patients is essential due to their increased susceptibility to TAC toxicity.

## Figures and Tables

**Table 1:** Demographic and clinical features of kidney transplant recipients.

Variables	*n* = 46
Age (years) *	29.8 ± 5.3
Sex **	
- Male - Female	36 (80) 10 (20)
BMI (kg/m^2^) *	24.7 ± 4.1
Evolution time of CKD (years) ***	4 (2–10)
Etiology of CKD **	
- Undetermined - Focal segmental glomerulonephritis - Lupus nephritis	43 (93.5) 2 (4.3) 1 (2.2)
RRT pre-KT **	
- PD - HD - None	19 (41.3) 22 (47.8) 5 (10.9)
Other comorbidities **	
- HBP - None	38 (82.6) 8 (17.4)
KT number **	
- First - Second	45 (97.8) 1 (2.2)
Donor type **	
- Live related - Live affective - Deceased donor	34 (73.9) 6 (13.04) 6 (13.04)
Type of immune induction pre-KT **	
- Basiliximabd - Thymoglobulin	31 (67.4) 15 (32.60)
Maintenance immunosuppression **	
- TAC-MPA-PDN	46 (100)
Other drugs	
- Omeprazole	11 (23.9)
- Calcium channel inhibitors	27 (58.7)

Abbreviations: BMI, body mass index; CKD, chronic kidney disease; RRT, renal placement therapy; KT, kidney transplant; PD, peritoneal dialysis; HD, hemodialysis; HBP, high blood pressure; TAC, tacrolimus; MPA, mycophenolic acid; PDN, prednisone. *: variables expressed as mean ± standard deviation. **: variables expressed as *n* (%). ***: Variables expressed as median (min–max).

**Table 2:** Genotype and allele frequencies and genetic profile in 45 KTR.

SNPs		Genotype		*n* (%)	Allele		*n* (%)	*n*	
CYP3A4-392A/G		AA		37 (82)	A		82 (91)	45	
		AG		8 (18)	G		8 (9)		
		GG		0	-		-		
CYP3A5-6986A/G		AA		0	A		17 (18)	46	
		AG		17 (37)	G		75 (82)		
		GG		29 (63)	-		-		
ABCB1-3435C/T		CC		3 (7)	C		35 (40)	44	
		CT		29 (65)	T		53 (60)		
		TT		12 (27)	-		-		
Genetic profile									
	CYP3A4392 A/G		CYP3A5-6986 A/G			ABCB1-3435 C/T			*n* (%)
1	G		A			T			46 (50)
2	G		A			C			24 (26)
3	A		A			T			4 (4)
4	A		A			C			9 (9)
Others	–		–			–			9 (9)

Percentages were estimated using 46 patients in total, and a total of 92 were used for the alleles. The genetic profiles were constructed from the genotyping of three SNPs: CYP3A4 (rs2740574), CYP3A5 (rs776746), and ABCB1 (rs1045642).

**Table 3:** Comparison of TAC concentration and doses according to genotype.

		Baseline Determination				One Month Later Determination			
Gene SNPs	Genotype	TAC Doses ***	*p*-Value	TAC Concentration ***	*p*-Value	TAC Doses ***	*p*-Value	TAC Concentration ***	*p*-Value
**CYP3A4** **392A/G**	AA	0.12 (0.10–0.12)	0.98	8.8 (5.9–12.5)	0.30	0.11 (0.06–0.17)	0.98	13.90 (4.0–27.10)	0.77
	AG	0.12 (0.6–0.12)		7.45 (4.8–10.32)		0.12 (0.05–0.15)		12.25 (4.60–19.10)	
	GG	-		-		-		-	
**CYP3A5** **6986A/G**	AA	-	0.71	-	0.30	-	0.34	-	0.006
	AG	0.12 (0.10–0.12)		6.9 (5.0–9.5)		0.11 (0.08–0.17)		9.20 (4.00–27.10)	
	GG	0.12 (0.6–0.12)		9 (7.1–12.5)		0.11 (0.05–0.17)		14.60 (4.60–23.90)	
**ABCB1** **3435C/T**	CC	0.12 (0.12–0.12)	0.35	8.2 (6.6–10.7)	0.99	0.10 (0.08–0.15)	0.45	14.20 (6.20–15.10)	0.79
	CT	0.12 (0.6–0.12)		8.2 (6.6–10.7)		0.11 (0.05–0.17)		13.90 (4.00–27.10)	
	TT	0.12 (0.10–0.12)		9.9 (6.45–11.7)		0.12 (0.06–0.15)		13.75 (7.70–18.90)	

***: Variables expressed as median (min–max). TAC serum concentration are expressed in ng/dL; the TAC dose is expressed in mg/kg/day.

**Table 4:** Comparison of TAC concentration, TAC dose, with biochemical parameters and drug interaction. Baseline and one month follow-up.

	Baseline	One Month Follow-Up	*p*-Value
TAC concentration (ng/dL) ***	8.1(5.0–11.8)	13.5 (9.0–16.8)	<0.001
TAC dose (mg/kg/day) ***	0.12(0.12–0.12)	0.11 (0.10–0.13)	0.619
Hematocrit (%) *	32.9 ± 6.7	39.5 ± 4.4	<0.001
Albumin (g/dL) *	4.11 ± 0.5	4.42 ± 0.4	0.074
Creatinine (mg/dL) *	1.3 (1.1–1.4)	1.01 (0.82–1.3)	<0.001
Drug interaction **	No = 16 (35) Yes = 30 (65)	No = 28 (61) Yes = 18 (39)	0.019

TAC, tacrolimus. *p*-values ≤ 0.05 were considered statistically significant. Note: the statistical test used for serum TAC and albumin levels, as well as for TAC dose. *: variables expressed as mean ± standard deviation. **: variables expressed as *n* (%). ***: variables expressed as median (min–max).

**Table 5:** Assessment of the association of genetic parameters (SNPs), TAC concentration, TAC doses, biochemical parameters, and drugs.

	TAC Concentration		
Variables	Group 1: <15 ng/dL	Group 2: >15 ng/dL	*p*-Value
CYP3A4-392A/G **			
AA	31 (68.9)	6 (13.3)	0.578 ^a^
AG	8 (17.8)	0 (0)	
CYP3A5-6986A/G **			
AG	15 (32.6)	2 (4.3)	>0.99 ^a^
GG	25 (54.3)	4 (8.7)	
ABCB1-3435C/T **			
CC	3 (6.8)	0 (0)	0.239 ^a^
CT	23 (52.3)	6 (13.6)	
TT	12 (27.3)	0 (0)	
TAC serum (ng/dL) * concentration **	7.35 (5.0–10.32)	21.95 (17.6–26.6)	<0.001 ^b^
TAC dose (mg/kg/day) ***	0.12 (0.12–0.12)	0.12 (0.12–0.12)	0.869 ^b^
Serum creatinine (mg/dL) ***	1.32 (1.0975–1.43)	1.195 (1.10–1.39)	0.935 ^b^
Hematocrit (%) ***	31.95 (27.60–36.27)	40.75 (37.75–43.82)	0.005 ^b^
Serum albumin (g/L) ***	4.0 (3.7–4.5)	4.20 (4.2–4.4)	0.371 ^b^
Hypoalbuminemia **			
Yes	3 (10)	0 (0)	>0.99 ^a^
No	22 (73.3)	5 (16.7)	
Use of drugs that elevate serum TAC concentration **	25 (54.3)	5 (10.9)	0.649 ^a^
Yes	15 (32.6)	1 (2.2)	
No			
One-month follow-up			
CYP3A4-392A/G **			
AA	24 (53.3)	13 (28.9)	0.452 ^a^
AG	4 (8.9)	4 (8.9)	
CYP3A5-6986A/G **			
AG	14 (30.4)	3 (6.5)	0.05 ^a^
GG	15 (32.6)	14 (30.4)	
ABCB1-3435C/T **			
CC	2 (4.5)	1 (2.3)	>0.99 ^a^
CT	18 (40.9)	11 (25.0)	
TT	7 (15.9)	5 (11.4)	
TAC serum (ng/dL concentration) ***	8.10 (4.00–11.90)	16.48 (12.00–27.10)	<0.001 ^b^
TAC dose (mg/kg/day) ***	0.11 (0.05–0.17)	0.12 (0.07–0.15)	0.598 ^b^
Serum creatinine (mg/dL) *	0.98 (±0.23)	1.16 (±0.36)	0.039 ^c^
Hematocrit (%) *	38.9 (±4.11)	40.5 (±4.78)	0.237 ^c^
Serum albumin (g/L) *	4.21 (±0.31)	4.59 (±0.32)	0.034 ^c^
Hypoalbuminemia:			
Yes	0 (0)	0 (0)	---
No	7 (43.75)	9 (56.25)	
Use of drugs that elevate serum TAC concentration:			
Yes	8 (17.4)	10 (21.7)	0.060 ^a^
No	21 (45.7)	8 (15.2)	

* variables expressed as mean ± standard deviation. ** variables expressed as *n* (%). *** variables expressed as median (min–max). ^a^: Mann–Whitney U test; ^b^: Chi-squared test or Fisher exact test. ^c^: student’s *t*-test. At baseline, 40 patients were identified with TAC >15 concentration ng/dL. After one month, 9 patients were identified with TAC concentration >15 ng/dL.

**Table 6:** Association of variables with TAC concentration >15 ng/mL at one-month follow-up.

Characteristics One Month Later	Odds Ratio (OR)	95% CI	*p*-Value
CYP3A5-6986A/G PM *	4.35	1.13–21.90	0.0458
CYP3A5-6986A/G PM **	9.32	1.54–93.08	0.028
Serum creatinine (mg/dL)	7.05	0.45–177.12	0.191
Hematocrit	1.07	0.90–1.28	0.439
Drug use that increases TAC concentration ^‡^	9.53	1.79–88.23	0.018

* individual evaluation of the CYP3A5-6986A/G polymorphism (PM); ** integrated evaluation of the CYP3A5-6986A/G polymorphism in the multivariate logistic regression model; ^‡^ drugs included: omeprazole and calcium channel inhibitors (amlodipine or nifedipine).

## Data Availability

Data are contained within the article.

## References

[1] Couser W., Remuzzi G., Mendis S., Tonelli M. (2011). The contribution of chronic kidney disease to the global burden of major noncommunicable diseases. Kidney Int..

[2] Robles-Osorio M.L., Sabath-Silva E., Sabath E. (2015). Arsenic-mediated nephrotoxicity. Ren. Fail..

[3] United States Renal Data System Annual Data Report [Internet]. USRDS. 2022 Annual Data Report: Atlas of Chronic Kidney Disease and End Stage Renal Disease in the US, NIH, NIDDKD. https://usrds-adr.niddk.nih.gov/2022.

[4] Pecoits R., Rosa G., Gonzalez M., Marinovich S., Fernandez S., Lugon J., Poblete H., Elgueta S., Gomez R., Cerdas M. (2015). Renal replacement therapy in CKD: An update from the Latin American Registry of Dialysis and Transplantation. J. Bras. Nefrol..

[5] Flagg A. (2018). Chronic Renal Therapy. Nurs. Clin. N. Am..

[6] Camilleri B., Pararajasingam R., Buttigieg J., Halawa A. (2020). Immunosuppression strategies in elderly renal transplant recipients. Transplant. Rev..

[7] Szumilas K., Wilk A., Wiśniewski P., Gimpel A., Dziedziejko V., Kipp M., Pawlik A. (2023). Current Status Regarding Immunosuppressive Treatment in Patients after Renal Transplantation. Int. J. Mol. Sci..

[8] Kapturczak M., Meier H., Kaplan B. (2004). Pharmacology of calcineurin antagonists. Transplant. Proc..

[9] McKeon F. (1991). When worlds collide: Immunosuppressants meet protein phosphatases. Cell.

[10] Hsiao C., Ho M., Ho C., Wu Y., Lee P., Hu R. (2021). Long-Term Tacrolimus Blood Trough Level and Patient Survival in Adult Liver Transplantation. J. Pers. Med..

[11] Wallemacq P., Verbeeck R. (2001). Comparative clinical pharmacokinetics of tacrolimus in paediatric and adult patients. Clin. Pharmacokinet..

[12] Christians U., Braun F., Schmidt M., Kosian N., Schiebel H., Ernst L., Winkler M., Kruse C., Linck A., Sewing K.F. (1992). Specific and sensitive measurement of FK506 and its metabolites in blood and urine of liver-graft recipients. Clin. Chem..

[13] Van T., Etsouli O., Moes J., Swen J. (2020). Comparison of the impact of pharmacogenetic variability on the PK of slow release and immediate release tacrolimus formulations. Genes.

[14] Cheng F., Li Q., Wang J., Hu M., Zeng F., Wang Z., Zhang Y. (2021). Genetic polymorphisms affecting tacrolimus metabolism and the relationship to post-transplant outcomes in kidney transplant recipients. Pharmgenomics Pers. Med..

[15] Yang M., Huan G., Wang M. (2018). Influence of CYP3A and ABCB1 Single Nucleotide Polymorphisms on the Pharmacokinetics/Pharmacodynamics of Tacrolimus in Pediatric Patients. Curr. Drug Metab..

[16] Ebid A., Ismail A., Lotfy N., Mahmoud M., ELSharkawy M. (2022). Influence of CYP3A4*22 and CYP3A5*3 combined genotypes on tacrolimus dose requirements in Egyptian renal transplant patients. J. Clin. Pharm. Ther..

[17] Janssens A.C., Moonesinghe R., Yang Q., Steyerberg E.W., van Duijn C.M., Khoury M.J. (2007). The impact of genotype frequencies on the clinical validity of genomic profiling for predicting common chronic diseases. Genet. Med..

[18] Favela A., Rangel H., Fricke I., Ortega A., Martínez G., López M. (2018). Genetic variability among Mexican Mestizo and Amerindian populations based on three ABCB1 polymorphisms. Mol. Biol. Rep..

[19] García P., Medeiros M., Reyes H., Rodríguez B., Alber J., Ortiz L., Vásquez M., Elizondo G., Morales L., Mancilla E. (2012). CYP3A5 polymorphism in Mexican renal transplant recipients and its association with tacrolimus dosing. Arch. Med. Res..

[20] MacPhee I. (2012). Pharmacogenetic biomarkers: Cytochrome P450 3A5. Clin. Chim. Acta.

[21] Velickovic R., Mikov M., Catic A., Stefanovic N., Stojanovic M., Jokanovic M., Cvetkovic T. (2012). Tacrolimus as a part of immunosuppressive treatment in kidney transplantation patients: Sex differences. Gend. Med..

[22] Evans E., McLeod H. (2003). Pharmacogenomics—Drug disposition, drug targets, and side effects. N. Engl. J. Med..

[23] Hamadeh I., Zhang Q., Steuerwald N., Hamilton A., Druhan L., McSwain M., Diez Y., Rusin S., Han Y., Symanowski J. (2019). Effect of CYP3A4, CYP3A5, and ABCB1 Polymorphisms on Intravenous Tacrolimus Exposure and Adverse Events in Adult Allogeneic Stem Cell Transplant Patients. Biol. Blood Marrow Transplant..

[24] Marsh S., King C.R., Van Booven D.J., Revollo J.Y., Gilman R.H., McLeod H.L. (2015). Pharmacogenomic assessment of Mexican and Peruvian populations. Pharmacogenomics.

[25] Miedziaszczyk M., Idasiak I. (2023). Safety analysis of co-administering tacrolimus and omeprazole in renal transplant recipients—A review. Biomed. Pharmacother..

[26] Miller S., Dykes D., Polesky H. (1988). A simple salting out procedure for extracting DNA from human nucleated cells. Nucleic Acids Res..

[27] Anglicheau D., Flamant M., Schlageter M., Martinez F., Cassinat B. (2003). Pharmacokinetic interaction between corticosteroids and tacrolimus after renal transplantation. Nephrol. Dial. Transplant..

[28] Schiff J., Cole E., Cantarovich M. (2007). Therapeutic monitoring of calcineurin inhibitors for the nephrologist. Clin. J. Am. Soc. Nephrol..

[29] Jouve T., Noble J., Rostaing L., Malvezzi P. (2019). An update on the safety of tacrolimus in kidney transplant recipients, with a focus on tacrolimus minimization. Expert Opin. Drug Saf..

[30] Cavalli S., Hirata M., Hirata R. (2001). Detection of MboII polymorphism at the 5′ promoter region of CYP3A4. Clin. Chem..

[31] van Schaik R.H.N., van der Heiden I.P., van den Anker J.N., Lindemans J. (2002). CYP3A5 variant allele frequencies in Dutch Caucasians. Clin. Chem..

[32] Sy S., Singh P., Shilbayeh S., Zmeili R., Conrado D., Derendorf H. (2013). Influence of CYP3A5 6986A>G and ABCB1 3435C >T Polymorphisms on Adverse Events Associated with Tacrolimus in Jordanian Pediatric Renal Transplant Patients. Clin. Pharmacol. Drug Dev..

[33] Cheng X., Chen Y., Zhang L., Chen B., Yang D., Chen W., Zhu P., Fang Z., Chen Z. (2022). Influence of *CYP3A5*, *IL-10* polymorphisms and metabolism rate on tacrolimus exposure in renal post-transplant recipients. Pharmacogenomics.

[34] Fathy M., Kamal M., Mohy A., Nabil A. (2016). Impact of CYP3A5 and MDR-1 gene polymorphisms on the dose and level of tacrolimus among living-donor liver transplanted patients: Single center experience. Biomarkers.

[35] Macphee I., Fredericks S., Mohamed M., Moreton M., Carter N., Johnston A., Goldberg L., Holt D. (2005). Tacrolimus pharmacogenetics: The CYP3A5*1 allele predicts low dose-normalized tacrolimus blood concentrations in whites and South Asians. Transplantation.

[36] Hesselink D.A., van Schaik R.H., Van Der Heiden I.P., van der Werf M., Gregoor P.J.H.S., Lindemans J., Weimar W., van Gelder T. (2003). Genetic polymorphisms of the CYP3A4, CYP3A5, and MDR-1 genes and pharmacokinetics of the calcineurin inhibitors cyclosporine and tacrolimus. Clin. Pharmacol. Ther..

[37] Roy J., Barama A., Poirier C., Vinet B., Roger M. (2006). Cyp3A4, Cyp3A5, and MDR-1 genetic influences on tacrolimus pharmacokinetics in renal transplant recipients. Pharmacogenet. Genom..

[38] Marín A., Gómez J., Mendoza N., Figuera L. (2023). Association between the Polymorphisms rs2070744, 4b/a and rs1799983 of the *NOS3* Gene with Chronic Kidney Disease of Uncertain or Non-Traditional Etiology in Mexican Patients. Medicina.

[39] Wesseling C., Crowe J., Hogstedt C., Jakobsson K., Lucas R., Wegman D. (2013). The epidemic of chronic kidney disease of unknown etiology in Mesoamerica: A call for interdisciplinary research and action; García, C. Importance of Pharmacogenetics and Drug-Drug Interactions in a Kidney Transplanted Patient. Am. Public Health.

[40] Staatz C., Tett S. (2004). Clinical pharmacokinetics and pharmacodynamics of tacrolimus in solid organ transplantation. Clin. Pharmacokinet..

[41] Sapir R., Wang Y., Famure O., Li Y., Kim S. (2014). Time-dependent variability in tacrolimus trough blood levels is a risk factor for late kidney transplant failure. Kidney Int..

[42] Størset E., Holford N., Midtvedt K., Bremer S., Bergan S., Åsberg A. (2014). Importance of hematocrit for a tacrolimus target concentration strategy. Eur. J. Clin. Pharmacol..

[43] Mourad M., Wallemac P., De Meyer M., Malaise J., De Pauw L., Eddour D., Goffin E., Lerut J., Haufroid V. (2008). Biotransformation enzymes and drug transporters pharmacogenetics in relation to immunosuppressive drugs: Impact on pharmacokinetics and clinical outcome. Transplantation..

[44] Katsakiori P., Papapetrou E., Goumenos D., Nikiforidis G., Flordellis C. (2010). Investigation of clinical interaction between omeprazole and tacrolimus in CYP3A5 non-expressors, renal transplant recipients. Ther. Clin. Risk Manag..

[45] Concha J., Sangüesa E., Saez A., Aznar I., Berenguer N., Saez L., Ribate M. (2023). Importance of Pharmacogenetics and Drug–Drug Interactions in a Kidney Transplanted Patient. Life.

[46] Williams P., Cosme J., Ward A., Angove H., Matak D., Jhoti H. (2003). Crystal structure of human cytochrome P450 2C9 with bound warfarin. Nature.

[47] Zhao W., Baudouin V., Fakhoury M., Storme T., Deschênes G., Jacqz-Aigrain E. (2012). Pharmacokinetic interaction between tacrolimus and amlodipine in a renal transplant child. Transplantation.

